# Identification and validation of QTLs for seedling salinity tolerance in introgression lines of a salt tolerant rice landrace ‘Pokkali’

**DOI:** 10.1371/journal.pone.0175361

**Published:** 2017-04-07

**Authors:** Teresa B. De Leon, Steven Linscombe, Prasanta K. Subudhi

**Affiliations:** 1 School of Plant, Environmental, and Soil Sciences, Louisiana State University Agricultural Center, Baton Rouge, LA, United States of America; 2 Rice Research Station, Louisiana State University Agricultural Center, Rayne, LA, United States of America; Pennsylvania State University, UNITED STATES

## Abstract

Salinity is a major threat to rice production worldwide. Several studies have been conducted to elucidate the molecular basis of salinity tolerance in rice. However, the genetic information such as quantitative trait loci (QTLs) and molecular markers, emanating from these studies, were rarely exploited for marker-assisted breeding. To better understand salinity tolerance and to validate previously reported QTLs at seedling stage, a set of introgression lines (ILs) of a salt tolerant donor line ‘Pokkali’ developed in a susceptible high yielding rice cultivar ‘Bengal’ background was evaluated for several morphological and physiological traits under salt stress. Both SSR and genotyping-by-sequencing (GBS) derived SNP markers were utilized to characterize the ILs and identify QTLs for traits related to salinity tolerance. A total of eighteen and thirty-two QTLs were detected using SSR and SNP markers, respectively. At least fourteen QTLs detected in the RIL population developed from the same cross were validated in IL population. Analysis of phenotypic responses, genomic composition, and QTLs present in the tolerant ILs suggested that the mechanisms of tolerance could be Na^+^ dilution in leaves, vacuolar Na^+^ compartmentation, and possibly synthesis of compatible solutes. Our results emphasize the use of salt injury score (SIS) QTLs in marker-assisted breeding to improve salinity tolerance. The tolerant lines identified in this study will serve as improved breeding materials for transferring salinity tolerance without the undesirable traits of Pokkali. Additionally, the lines will be useful for fine mapping and map-based cloning of genes responsible for salinity tolerance.

## Introduction

Backcrossing is an established and efficient approach in introgression of both qualitative and quantitative traits from landraces and wild relatives to elite adapted varieties. The use of advanced backcross populations or introgression lines (ILs) has been widely employed in genetic studies to identify and validate the beneficial effects of QTLs from donor parents [1]. In tomato, ILs were useful in fine mapping of QTLs for fruit mass [2]. Likewise, ILs were developed and used in QTL mapping for fusarium head blight resistance in wheat [3], mineral accumulation in beans [4], yield attributes in soybean [5], and nematode and fusarium wilt disease resistance in cotton [6]. In rice, several introgression line populations were developed to transfer and map QTLs for agronomic and domestication traits [7–8], yield and morphological traits [9–11], Zn and Fe content in grain [12], and photosynthesis parameters [13].

Among the abiotic stresses, soil and water salinity is a major crop production constraint in the arid regions and coastal areas that heavily relied on irrigation. The genetics of salinity tolerance in rice has been investigated for many years. Several QTLs and genes for morphological and physiological traits associated with salinity tolerance were reported [14–19]. However, application of QTLs and molecular markers for development of salt tolerant rice varieties is still difficult and slow [20]. The majority of QTLs detected so far in various mapping populations were small effect QTLs that were neither validated nor exploited to improve salinity tolerance in breeding programs. Furthermore, the well-known and widely used tolerant donors, Pokkali and Nona Bokra, are low yielding and possess many undesirable agronomic traits that complicate the breeding process. They are tall, susceptible to lodging, sensitive to photoperiod, and the grains are awned with red pericarp [21]. To address the linkage drag associated with landraces, and for discovery of genes responsible for abiotic and biotic tolerance, the International Rice Research Institute (IRRI) had initiated a backcross breeding program in which 203 donor accessions were crossed to three high yielding varieties as recurrent parents [22]. After 4 cycles of backcrossing, screening, and progeny testing, large number of introgression lines with significantly improved tolerance to biotic and abiotic stress were generated. Genotyping of selected 83 ILs using 160 SSR markers allowed the discovery and fine mapping of QTL for drought tolerance to a small region of ~3cM [23]. For salinity, backcross lines derived from Pokkali were evaluated to validate the *Saltol* QTL [24]. However, further studies are needed because backcross lines containing *Saltol* and non-*Saltol* QTL showed the same level of seedling salinity tolerance. Moreover, evaluation of near isogenic lines containing *Saltol* locus in the field under salt stress did not show higher yield performance than the susceptible IR29 [17].

The need for QTLs and molecular markers predictive of salinity tolerance is still a challenge. For these reasons, it is important to confirm the stability and the contribution of QTLs toward salinity tolerance. Most of the QTL mapping studies were implemented in F_2:3_ and RIL populations with a limited number of genotypes and markers. In this study, we used ILs for QTL mapping of nine traits related to salinity tolerance using SSR and GBS-derived SNP markers. The QTLs identified in the ILs were compared to previously mapped QTLs in the RIL population developed from the same cross for confirmation. Also, we identified salinity tolerant lines that were near isogenic to Bengal which would be useful as improved variety or resource materials in transferring salinity tolerance genes to other elite US varieties.

## Materials and methods

### Plant material and evaluation for salt tolerance

Introgression lines were developed from a cross between Pokkali and Bengal. Pokkali is highly tolerant to salinity stress [21] while Bengal is highly salt sensitive [25]. Bengal and Pokkali were used as recurrent and donor parent, respectively. Due to pollen sterility of F_1_ plants, Bengal was used as pollen parent to generate BC_1_ generation. However, in BC_1_ and subsequent backcross generation Bengal was used as female parent to generate the BC_4_F_1_ population which was then self-pollinated repeatedly to finally produce BC_4_F_4_ lines by single seed descent method.

A total of 292 BC_4_F_4_ lines were screened for seedling salinity tolerance following the protocol described by De Leon et al. [25]. Briefly, ten plants per line per replication were grown for two weeks in nutrient solution containing 1g/L of Jack’s Professional fertilizer 20-20-20 (J.R. Peters Inc.) and 300mg/L ferrous sulfate. The seedlings were then placed in nutrient solution containing NaCl at salt stress level of 6 dSm^-1^ for two days before subjecting to 12 dSm^-1^ salt stress. Only five plants of uniform growth were scored for morphological and physiological traits related to salinity tolerance. Chlorophyll content (CHL) was measured using a SPAD-502 chlorophyll meter (Spectrum Technologies, Inc.) four days after salt stress. When the susceptible parent Bengal showed the characteristic salt sensitivity reaction, plants were scored for visual salt injury score (SIS) of 1 to 9. ILs that showed normal growth similar to their corresponding lines grown in control were scored 1. A score of 3 was given to plants that showed normal growth but stunted compared to control plants. A score of 5 was given to plants that were stunted, with green rolled leaves and few whitish tips. When plants showed dried leaves but with green culms, a score of 7 was given while completely dead and dried plants were scored 9.The root and shoot lengths (RTL, SHL) were measured during this time. Shoot length was measured in cm from the base of the culm to the tip of the longest leaf while root length was measured from the base of the culm to the tip of the root mass. The ratio of shoot length to root length (SRR) were computed while shoot dry weight (DWT) data were obtained from five plants per line that were oven-dried at 65°C oven for five days prior to weighing. The concentrations of Na^+^ and K^+^ in the shoots were estimated from 100 mg tissue taken from a pool of five oven-dried plants. The ground tissues were digested by nitric acid: hydrogen peroxide (5:3 ml) method at 152–155°C heating block for 3 hours [26]. The amount of Na^+^ and K^+^ was measured by flame photometer (model PFP7, Bibby Scientific Ltd, Staffordshire, UK). The final concentrations of Na^+^ and K^+^ ions were estimated using the standard curve derived from different dilutions. The whole experiment was conducted in randomized complete block design replicated three times.

### Statistical analyses

Analysis of variance for each trait was computed by Glimmix procedure where the line was entered as fixed effect and replication was entered as random effect. Least square means of each line was extracted for QTL analysis. To see the relationship among traits, correlation procedure was employed. All data analysis was conducted using Statistical Analysis System (SAS) software version 9.4 for Windows [27]. Histograms were constructed in Microsoft Excel 2010 to show the distribution of introgression lines for each phenotypic trait.

### Genotyping of ILs using SSR and SNP markers

Leaf tissues from 292 lines were collected from each BC_4_F_4_ line grown in non-saline nutrient solution. The tissues were ground and genomic DNAs were isolated following the CTAB method [28]. The concentration of each DNA was estimated by a ND-1000 spectrophotometer (Thermo Fisher Scientific, Wilmington, USA) and was adjusted to 25ng/μl for PCR amplification. A total of 136 SSR primers were surveyed for polymorphism between parents but only 107 polymorphic SSR markers were used for initial genotyping of the population (S1 Table). For each PCR reaction, the mixture contained 12.8μl water, 2.5μl 10X PCR buffer, 2.5μl 25mM MgCl_2_, 2.5μl 2mM dNTPs, 1.25μl reverse and forward primers (50ng/μl), 1U Taq polymerase (Promega Corporation, Madison, USA) and 50 ng of DNA. The PCR amplification was conducted with the following settings: initial denaturation at 94°C for 5 min, 35 cycles of 94°C for 45 sec, 55°C for 45 sec, 72°C for 1 min and a final extension at 72°C for 5 min. The PCR products were run in 4.5% SFR agarose gel electrophoresis and alleles of each line were scored according to the banding pattern of the parents. From the 292 lines, a subset of 88 lines with varying levels of salinity response based on SIS and introgressions were randomly selected for genotyping-by-sequencing (GBS). The DNA from 88 ILs were extracted using the Qiagen DNeasy Plant Mini Kit following the manufacturer’s protocol (Qiagen Inc., Valencia, CA, USA). The Genomic Diversity Facility, Cornell University Institute of Biotechnology (http://www.biotech.cornell.edu/brc/genomic-diversity-facility) provided the GBS service that included the genomic DNA library construction following the method of Elshire et al. [29], 288-plex sequencing using the Illumina HiSeq sequencer, and SNP calling based on the Nipponbare reference genome MSU release 7 [30]. The resultant GBS data were filtered for QTL analysis. Each SNP call at a particular coordinate was treated as a marker. Due to low read depth in GBS, all heterozygous SNP calls were treated as missing data. All non-polymorphic SNP markers across the 88 introgression lines were removed. Likewise, all SNP markers having more than 10% missing data or N calls were discarded before further analysis. A total of 6,797 SNP markers were retained and used (S2 Table). The SNP calls for each line were scored as either Pokkali or Bengal allele.

### Estimation of genome composition and QTL analysis for traits related to salinity tolerance

The genotypic data using SSR and GBS-SNP markers were used separately to estimate the genome composition of each line. The physical position of SSR markers along the chromosomes were obtained from Gramene website (www.gramene.org) while SNP markers were ordered based on their physical positions in the rice genome (MSU release 7). The length of introgressed segments in each IL was estimated based on graphical genotypes [31]. If alleles of the two adjacent markers were the same, the chromosome segment was assumed entirely of that marker genotype. If two consecutive markers showed different alleles in a chromosome segment, the interval was divided equally among both the markers. These estimates disregard the possibility of double recombinants within that interval. Genotypes were selected to represent a set of chromosome segment substitution lines (CSSL) or ILs using CSSL finder v. 0.9.7.2.2 [32]. Percent genome composition and introgressed Pokkali segments of each IL were computed from the CSSL analysis.

The phenotypic and genotypic data were combined and used in the CSSL QTL mapping function of QTL IciMapping software v. 4.1 [33]. By single marker analysis (SMA) and stepwise regression-based likelihood ratio test (LRT) methods, significant QTLs were identified at LOD threshold set at 2.0. Significant QTL was named by the name of the trait followed by the chromosome number and the Mb position of the QTL along the chromosome. For example, *qSIS9*.*8* denotes a SIS QTL on chromosome 9 at 8 Mb region; *qSIS5*.*034* denotes a SIS QTL on chromosome 5 at 340 Kb region while *qSHL1*.*3810* and *qSHL1*.*3818* correspond to SHL QTLs on chromosome 1 at 38.10 and 38.18 Mb regions. The position and the effect of QTLs were estimated. To validate the effects and significance of QTLs for each trait, the positions of QTLs detected in ILs were compared to those QTLs detected earlier in Bengal x Pokkali RIL population [19]. Introgression lines with high salt tolerance were selected for further evaluation of genomic composition, phenotypic attributes, and QTLs they contained for inquiry of possible tolerance mechanisms.

## Results

### Phenotypic evaluations

The trait responses of ILs and the parents under salt stress were summarized in Table 1. There were significant differences between Bengal and Pokkali for NaK, SIS, SHL, RTL, DWT, and SRR. However, the difference in Na^+^, K^+^ concentrations, and CHL were not statistically significant. In the 292 ILs, significant phenotypic differences were observed for all traits except in CHL. The spread of trait means indicated the presence of transgressive segregants (S1 Fig). In all traits, the distributions of ILs were continuous and close to normal distribution.

**Table 1:** Mean phenotypic response of parents and 292 ILs (BC_4_F_4)_ under salt stress.

Trait Name	Bengal Mean	Pokkali Mean^$^	ILs			
			Mean	Std. Dev.	P>F^€^	Range
Na^+^ (mmolkg^-1^)	1232.57	940.82^ns^	1277.47	295.95	0.2804	859.34–1821.31
K^+^ (mmolkg^-1^)	548.08	590.19^ns^	575.48	159.75	<0.0001	320.93–836.66
NaK (ratio)	2.29	1.59*	2.31	0.57	<0.0001	1.62–3.92
SIS	7.80	3.00***	6.60	1.21	<0.0001	3.22–9.00
CHL (SPAD unit)	19.00	16.05^ns^	22.17	6.61	0.2136	15.85–45.32
SHL (cm)	31.67	47.20**	33.11	5.39	<0.0001	22.40–55.80
RTL (cm)	8.68	9.97*	8.77	1.00	<0.0001	4.05–10.37
DWT (g)	0.07	0.14**	0.08	0.02	<0.0001	0.048–0.133
SRR (ratio)	3.66	4.75**	3.81	0.70	<0.0001	2.91–6.80

^$^ t-test between Bengal and Pokkali Means

^ns^not significantly different

*significant at α = 0.05

**significant at α = 0.01

***significant at α = 0.001.

^€^ Genotypic differences among ILs. Na^+^: shoot sodium concentration, K^+^: shoot potassium concentration, NaK: ratio of the shoot sodium and shoot potassium content, SIS: salt injury score, CHL: chlorophyll content, SHL: shoot length, RTL: root length, DWT: shoot dry weight, SRR: shoot length to root length ratio.

The shoot Na^+^ concentration was significant and positively correlated to shoot K^+^ concentration, NaK, and SIS. Shoot Na^+^ concentration was also positively correlated to CHL (Table 2), which is probably due to lack of significant differences among ILs. On the other hand, SIS was significant and negatively correlated to SHL, RTL, SRR, and DWT. The relationship between shoot K^+^ concentration and NaK ratio was highly significant but negatively correlated. RTL, SRR and DWT were all significantly and positively correlated to SHL.

**Table 2:** Pearson correlation matrix of traits under seedling salinity stress in 292 ILs.

	Na^+^	K^+^	NaK	SIS	CHL	SHL	RTL	SRR	DWT
Na^+^	1								
K^+^	0.592***	1							
NaK	0.129*	-0.698***	1						
SIS	0.173**	0.134*	-0.088	1					
CHL	0.144*	0.197***	-0.117*	0.040	1				
SHL	-0.019	-0.050	0.083	-0.381***	-0.060	1			
RTL	0.077	0.143*	-0.107	`-0.118*	0.123*	0.337***	1		
SRR	-0.084	-0.146*	0.139*	-0.266***	-0.146*	0.680***	-0.435***	1	
DWT	-0.110	-0.185**	0.188**	-0.636***	0.029	0.564***	0.314***	0.291***	1

*significant at α = 0.05

**significant at α = 0.01

***significant at α = 0.001

Na^+^: shoot sodium concentration, K^+^: shoot potassium concentration, NaK: ratio of the shoot sodium and shoot potassium content, SIS: salt injury score, CHL: chlorophyll content, SHL: shoot length, RTL: root length, DWT: shoot dry weight, SRR: shoot length to root length ratio.

### Evaluation of genome composition and construction of ILs by SSR markers

**Fig 1:**
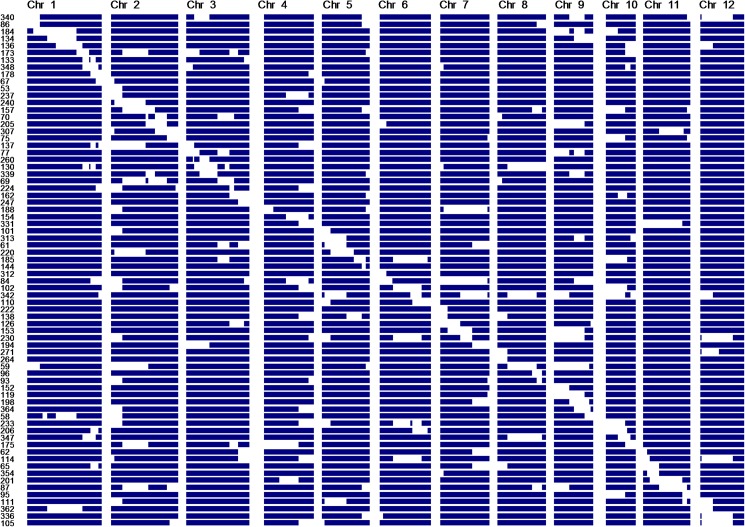
Graphical genotypes of 72 introgression lines developed from Bengal x Pokkali cross. Each row denotes a line selected for a chromosomal segment. Blue and white segments represent Bengal and Pokkali segments, respectively. Lines were genotyped using 107 SSR markers.

**Fig 2:**
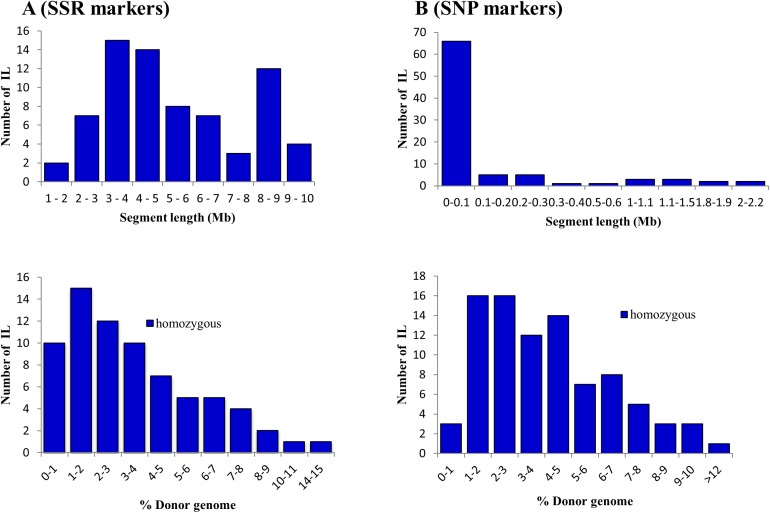
Frequency distribution of introgressed Pokkali segments (segment length and % donor genome) in the selected introgression lines using SSR (A) and SNP (B) markers.

A total of 107 markers were polymorphic between parents. These polymorphic markers were used to inquire the genetic make-up of ILs and for QTL mapping (S1 Table). The SSR markers were distributed over the rice genome every 3.7 Mb or every 15cM, with an average of 9 markers per chromosome (Table 3). Out of 292 BP BC_4_F_4_ lines that were phenotyped, only 276 lines had complete genotypic data for all SSR markers. Using the CSSL finder, the genome composition and Pokkali segments were evaluated in ILs. Out of 276 lines, 72 ILs were selected by the program to cover the 12 chromosomes of rice (Fig 1). A total of 216 segments covering about 77% of Pokkali genome were transmitted to the ILs. Each chromosome contained an average of 6 segments accounting to an average size of 5.3 Mb. Segments of Pokkali genome were fully (100%) represented in chromosomes 2 and 9. Chromosomes 8 and 12 had 50% and 57% coverage, respectively while other chromosomes had 66–68% coverage (Table 3). On average, the genome composition of each IL had 95% Bengal, with a minimum and maximum of 86% and 99%. In contrast, each IL contained an average introgression of 4.7%, with minimum of 0.8% and maximum of 14% Pokkali segments in selected introgression lines using SSR markers (S4 Table). The majority of ILs had 1–2% Pokkali segment with 3–5 Mb length (Fig 2A).

**Table 3:** Basic statistics of Pokkali segments in introgression lines using SSR and SNP markers.

	SSR Marker information			Introgression Lines^a^				SNP Marker information			Introgression Lines^b^			
Chr.	No. of markers used	Marker coverage (Mb)	Ave. Marker interval (Mb)	No. of Segments	Ave. % donor segment	Ave. donor segment size (Mb)	%Pokkali genome coverage^c^	No. of markers used	Marker coverage (Kb-Mb)	Ave. Marker interval (Kb)	No. of Segments	Ave. % donor segment	Ave. segment size (Mb)	%Pokkali genome coverage^c^
1	13	1–43	3.50	22	4.69	4.97	82	927	15–43	46.6	567	3.41	0.17	100
2	10	1–39	4.21	34	4.43	6.43	100	513	50–36	69.9	166	3.03	0.38	100
3	13	0.8–36	2.95	24	4.52	5.39	89	853	263–36	42.7	394	4.78	0.19	100
4	8	4–33	4.03	18	3.40	4.98	52	508	310–35	69.9	297	2.86	0.35	100
5	7	0.4–27	3.84	20	4.98	5.20	85	615	87–29	47.9	216	3.48	0.36	100
6	11	1.8–30	3.18	11	5.47	5.32	66	521	139–31	58.6	149	2.37	0.34	100
7	8	1–29	4.01	19	6.11	4.73	70	587	19–29	50.3	259	6.38	0.46	100
8	7	0.38–28	4.57	15	4.78	4.37	51	623	51–28	45.6	505	2.79	0.12	100
9	7	0.30–23	3.73	21	3.39	6.13	100	446	244–23	51.1	263	8.00	0.59	100
10	6	3–20	3.38	15	6.75	6.78	87	408	49–23	56.6	140	4.20	0.49	100
11	9	0–27	3.35	12	3.58	5.21	93	486	124–29	59.6	170	6.74	0.68	100
12	8	1–26	3.50	5	3.54	4.10	57	310	279–27	88.6	72	3.10	0.59	100
Sum	107	361		216		63.61	932	6797	370		3198		4.72	
Ave.	9	1–30	3.69	6	4.64	5.30	78	566	135–31	57.3	266	4.26	0.39	100

^a^ computed from 72 ILs genotyped by SSR markers.

^b^computed from 88 ILs genotyped by SNP markers.

^c^ computed from the proportion of homozygous Pokkali chromosome segment (in Mb) represented by at least one IL. Ave, Average.

### Evaluation of genome composition and construction of ILs by GBS-SNP markers

After filtering of the GBS data for the 88 ILs, a total of 6,797 SNP markers were retained and used for inquiry of genome composition and QTL mapping in ILs (S2 Table). An average of 566 SNP markers was placed in each chromosome with an average interval of 57.3 Kb between markers. The genome compositions of 88 ILs were summarized in S5 Table. On average, the genome composition of an IL was 95.8% Bengal and 4.1% Pokkali. Among the ILs, the number of Pokkali segments ranged from 6–143 segments that were distributed from one to twelve chromosomes of rice. Collectively, a total of 3,198 Pokkali segments were detected by SNP markers in the 88 ILs, with 266 segments per chromosome or an average size of 390 Kb segment per chromosome (Table 3). The high frequency of SNP markers per chromosome indicated whole genome coverage of Pokkali among ILs. Chromosome 1 and 12 contained the highest and lowest number of Pokkali segments, respectively. The majority of the ILs was carrying 1–3% Pokkali genome (Fig 2B).

### QTL analysis for traits related to salinity tolerance

**Fig 3:**
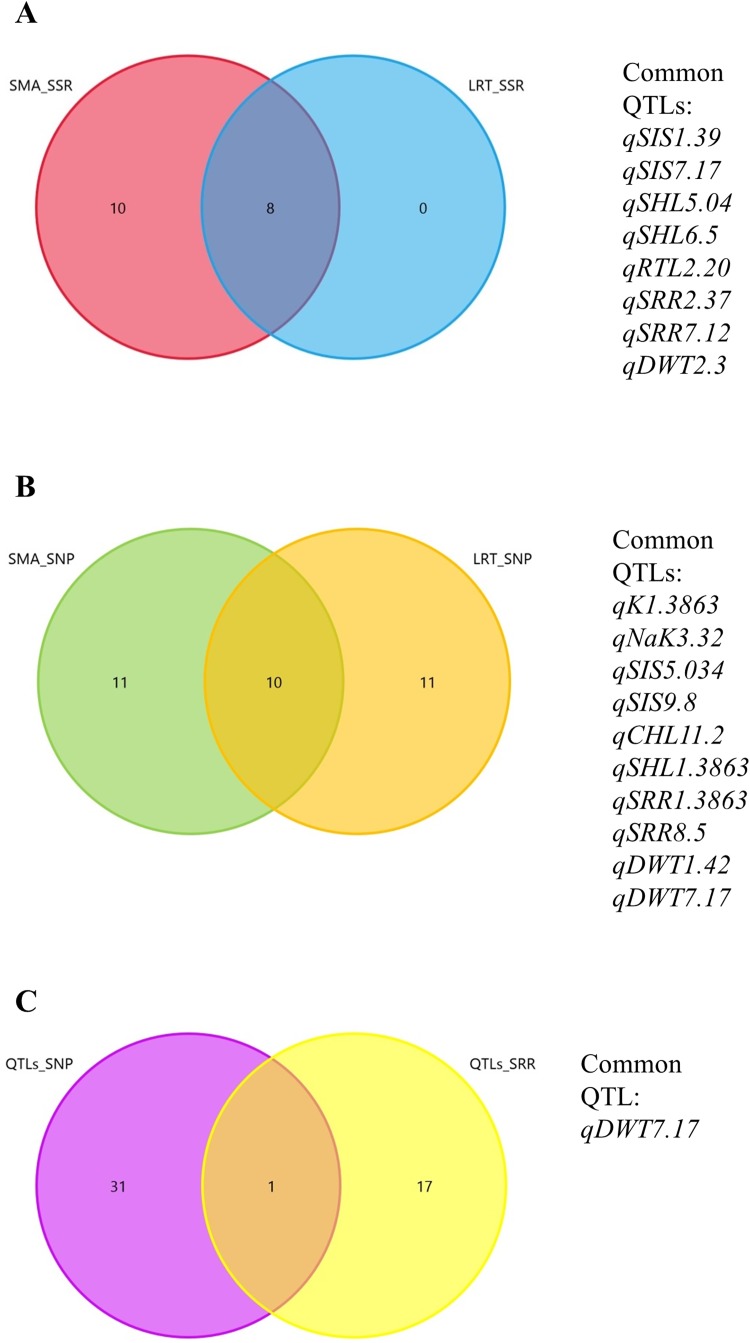
Comparison of salinity-tolerance QTLs detected in Pokkali introgression lines by single marker analysis (SMA) and stepwise regression-based likelihood ratio test (LRT). (A) Numbers of QTLs detected by SSR markers. (B) Number of QTLs detected by SNP markers. (C) Common QTL identified by SSR and SNP markers.

QTL analyses for nine traits were conducted separately in ILs genotyped by SSR and SNP markers. Single marker analysis (SMA) and stepwise regression-based likelihood ratio test (LRT) methods were employed to see the consistency of detecting QTLs. For QTL mapping in 72 ILs using SSR markers, a total of 18 QTLs were detected by SMA for five traits (Table 4) and 8 of these QTLs were also significant by LRT. There were no significant QTLs detected for shoot Na^+^, K^+^ concentrations, NaK, and CHL. In contrast, QTL mapping in 88 ILs using SNP markers detected a total of 32 QTLs for 8 traits (Table 5) and 10 QTLs were common and significant by SMA and LRT. Due to the differences in density and positions of SSR and SNP markers, only *qDWT7*.*17* was found similar in QTL mapping by SSR and SNP markers (Fig 3).

**Table 4:** QTLs detected in CSSLs by single marker analysis (SMA) and stepwise regression-based likelihood ratio test (LRT) using SSR markers.

Trait	QTL	CHR	Position (Mb)	Marker	LOD	PVE (%)	Additive Effect	Parental source of increasing allele^¥^	Lines containing Pokkali allele at QTL	Phenotype (mean value)
SIS_SMA	*qSIS1*.*39*	1	39.5	RM3810	2.275	6.456	-0.644	B	IL84	SIS(3.2)
	* *								IL178	SIS(5.4)
	* *								IL348	SIS(6.2)
	*qSIS2*.*3*	2	3	RM211	2.326	6.59	-0.442	B	IL84	SIS(3.2)
	* *								IL188	SIS(5.7)
	* *								IL137	SIS(5.8)
	* *								IL53	SIS(6.1)
	* *								IL101	SIS(6.2)
	* *								IL224	SIS(6.5)
	* *								IL230	SIS(4.1)
	* *								IL233	SIS(5.3)
	* *								IL59	SIS(5.3)
	*qSIS6*.*5*	6	5.4	RM253	2.498	7.04	-1.589	B	IL84	SIS(3.2)
	*qSIS7*.*12*	7	12.8	RM214	2.081	5.942	-0.855	B	IL84	SIS(3.2)
	* *								IL153	SIS(5.2)
	* *								IL188	SIS(5.7)
	*qSIS7*.*17*	7	17.5	RM5793	2.935	8.162	-0.788	B	IL84	SIS(3.2)
	* *								IL153	SIS(5.2)
	* *								IL188	SIS(5.7)
	* *								IL230	SIS(4.1)
	* *								IL342	SIS(6.2)
SIS_LRT	*qSIS1*.*39*	1	39.5	RM3810	2.055	10.154	-0.56	B	IL84	SIS(3.2)
	* *								IL178	SIS(5.4)
	* *								IL348	SIS(6.2)
	*qSIS7*.*17*	7	17.5	RM5793	2.935	16.989	-0.788	B	IL84	SIS(3.2)
	* *								IL153	SIS(5.2)
	* *								IL188	SIS(5.7)
	* *								IL230	SIS(4.1)
	* *								IL342	SIS(6.2)
SHL_SMA	*qSHL1*.*39*	1	39.5	RM3810	2.556	8.188	2.927	P	IL84	SHL(50cm)
	* *								IL178	SHL(39cm)
	* *								IL348	SHL(36cm)
	*qSHL1*.*41*	1	41.1	RM5362	2.091	6.795	2.899	P	IL178	SHL(39cm)
	* *								IL67	SHL(35cm)
	* *								IL137	SHL(36cm)
	* *								IL224	SHL(34cm)
	* *								IL8	SHL(50cm)
	*qSHL2*.*3*	2	3	RM211	2.031	6.614	1.788	P	IL84	SHL(50cm)
	* *								IL188	SHL(36cm)
	* *								IL137	SHL(36cm)
	* *								IL53	SHL(35cm)
	* *								IL101	SHL(35cm)
	* *								IL224	SHL(34cm)
	* *								IL230	SHL(38cm)
	* *								IL233	SHL(36cm)
	* *								IL59	SHL(34cm)
	*qSHL5*.*04*	5	0.4	RM17749	2.674	8.535	3.606	P	IL67	SHL(35cm)
	* *								IL101	SHL(35cm)
	* *								IL230	SHL(38cm)
	* *								IL105	SHL(53cm)
	*qSHL6*.*5*	6	5.4	RM253	4.152	12.67	8.603	P	IL84	SHL(50cm)
	*qSHL7*.*12*	7	12.8	RM214	2.067	6.722	3.669	P	IL84	SHL(50cm)
	* *								IL153	SHL(35cm)
	* *								IL188	SHL(36cm)
SHL_LRT	*qSHL5*.*04*	5	0.4	RM17749	3.861	16.175	3.734	P	IL67	SHL(35cm)
	* *								IL101	SHL(35cm)
	* *								IL230	SHL(38cm)
	* *								IL105	SHL(53cm)
	*qSHL6*.*5*	6	5.4	RM253	5.34	23.491	8.81	P	IL84	SHL(50cm)
RTL_SMA	*qRTL2*.*20*	2	20.7	RM341	2.203	12.976	-0.431	B	IL173	RTL(7.8cm)
	* *								IL157	RTL(8.3cm)
	* *								IL59	RTL(8.3cm)
	* *								IL175	RTL(7.7cm)
	* *								IL87	RTL(6.8cm)
RTL_LRT	*qRTL2*.*20*	2	20.7	RM341	2.203	12.976	-0.431	B	IL173	RTL(7.8cm)
	* *								IL157	RTL(8.3cm)
	* *								IL59	RTL(8.3cm)
	* *								IL175	RTL(7.7cm)
	* *								IL87	RTL(6.8cm)
SRR_SMA	*qSRR2*.*37*	2	37.6	RM266	3.057	18.445	0.409	P	IL102	SRR(4.26)
	* *								IL87	SRR(4.47)
	* *								IL105	SRR(5.83)
	*qSRR7*.*12*	7	12.8	RM214	2.034	12.665	0.389	P	IL84	SRR(5.14)
	* *								IL153	SRR(4.00)
	* *								IL188	SRR(4.70)
SRR_LRT	*qSRR2*.*37*	2	37.6	RM266	3.882	18.174	0.427	P	IL102	SRR(4.26)
	* *								IL87	SRR(4.47)
	* *								IL105	SRR(5.83)
	*qSRR7*.*12*	7	12.8	RM214	2.857	12.936	0.413	P	IL84	SRR(5.14)
	* *								IL153	SRR(4.00)
	* *								IL188	SRR(4.70)
DWT_SMA	*qDWT2*.*3*	2	3	RM211	5.192	14.881	0.01	P	IL84	DWT(0.133)
	* *								IL188	DWT(0.096)
	* *								IL137	DWT(0.094)
	* *								IL53	DWT(0.081)
	* *								IL101	DWT(0.091)
	* *								IL224	DWT(0.077)
	* *								IL230	DWT(0.111)
	* *								IL233	DWT(0.090)
	* *								IL59	DWT(0.082)
	*qDWT6*.*5*	6	5.4	RM253	2.921	8.966	0.028	P	IL84	DWT(0.133)
	*qDWT7*.*12*	7	12.8	RM214	2.357	7.362	0.015	P	IL84	DWT(0.133)
	* *								IL153	DWT(0.081)
	* *								IL188	DWT(0.096)
	*qDWT7*.*17*	7	17.5	RM5793	2.324	7.266	0.012	P	IL84	DWT(0.133)
	* *								IL153	DWT(0.081)
	* *								IL188	DWT(0.096)
	* *								IL230	DWT(0.111)
	* *								IL342	DWT(0.068)
DWT_LRT	*qDWT2*.*3*	2	3	RM211	5.192	27.93	0.01	P	IL84	DWT(0.133)
	* *								IL188	DWT(0.096)
									IL137	DWT(0.094)
									IL53	DWT(0.081)
									IL101	DWT(0.091)
									IL224	DWT(0.077)
									IL230	DWT(0.111)
									IL233	DWT(0.090)
									IL59	DWT(0.082)

^¥^Parental source of increasing allele: B, Bengal; P, Pokkali.

CHR: Chromosome; LOD: Logarithm of odds; PVE: Phenotypic variation explained by each QTL.

**Table 5:** QTLs detected in CSSLs by single marker analysis (SMA) and stepwise regression-based likelihood ratio test (LRT) using 6797 SNP markers.

Trait	QTL	CHR	Position (Mb)	Marker	LOD	PVE (%)	Additive Effect	Parental source of increasing allele^¥^	Lines containing Pokkali allele at QTL	Phenotype (mean value)
Na_LRT	*qNa11*.*5*	11	5.61	S11_5610372	2.04	9.68	-82.38	B	IL186	Na(938)
	* *								IL263	Na(1032)
	* *								IL262	Na(1080)
	* *								IL353	Na(1011)
	* *								IL57	Na(1111)
	* *								IL65	Na(1198)
	* *								IL89	Na(1253)
	* *								IL91	Na(1240)
K_SMA	*qK1*.*3863*	1	38.63	S1_38636497	2.23	10.66	69.26	P	IL178	K(753)
	* *								IL303	K(768)
	* *								IL323	K(748)
	* *								IL348	K(736)
	* *								IL51	K(518)
	* *								IL84	K(599)
K_LRT	*qK1*.*3863*	1	38.63	S1_38636497	2.23	10.66	69.26	P	IL178	K(753)
	* *								IL303	K(768)
	* *								IL323	K(748)
	* *								IL348	K(736)
	* *								IL51	K(518)
	* *								IL84	K(599)
NaK_SMA	*qNaK3*.*32*	3	32	S3_32078967	2.31	11.27	0.33	P	IL51	NaK(2.34)
	* *								IL52	NaK(3.64)
	* *								IL61	NaK(2.30)
	* *								IL98	NaK(2.83)
NaK_LRT	*qNaK3*.*32*	3	32	S3_32078967	2.31	11.27	0.33	P	IL51	NaK(2.34)
	* *								IL52	NaK(3.64)
	* *								IL61	NaK(2.30)
	* *								IL98	NaK(2.83)
SIS_SMA	*qSIS5*.*034*	5	0.34	S5_340482	2.79	7.14	-0.05	B	IL230	SIS(4.10)
	* *								IL313	SIS(5.13)
	* *								IL67	SIS(5.0)
	* *								IL68	SIS(4.47)
	* *								IL83	SIS(5.53)
	* *								IL91	SIS4.33
	* *								IL99	SIS(4.37)
	*qSIS5*.*1*	5	1.47	S5_1473882	2.36	5.53	-0.07	B	IL230	SIS(4.10)
	* *								IL313	SIS(5.13)
	* *								IL91	SIS(4.33)
	* *								IL99	SIS(4.37)
	*qSIS5*.*2*	5	2.83	S5_2831482	2.07	4.97	-0.05	B	IL230	SIS(4.10)
	* *								IL313	SIS(5.13)
	* *								IL61	SIS(5.07)
	* *								IL91	SIS(4.33)
	* *								IL99	SIS(4.37)
	*qSIS9*.*8*	9	8.6	S9_8608506	2.17	5.35	-0.05	B	IL116	SIS(4.40)
	* *								IL119	SIS(4.33)
	* *								IL230	SIS(4.10)
	* *								IL99	SIS(4.37)
	*qSIS9*.*14*	9	14.6	S9_14600108	2.18	5.25	-0.04	B	IL116	SIS(4.40)
	* *								IL119	SIS(4.33)
	* *								IL178	SIS(5.40)
	* *								IL63	SIS(5.53)
	* *								IL74	SIS(5.40)
	* *								IL84	SIS(3.22)
	* *								IL98	SIS(5.53)
	* *								IL99	SIS(4.37)
SIS_LRT	*qSIS1*.*41*	1	41.81	S1_41818521	2.05	6.17	-0.05	B	IL178	SIS(5.40)
	* *								IL323	SIS(5.65)
	* *								IL52	SIS(5.40)
	* *								IL67	SIS(5.00)
	* *								IL84	SIS(3.22)
	*qSIS1*.*42*	1	42.31	S1_42310908	2.27	6.8	-0.07	B	IL178	SIS(5.40)
	* *								IL67	SIS(5.00)
	* *								IL84	SIS(3.22)
	*qSIS5*.*034*	5	0.34	S5_340482	2.5	8.14	-0.05	B	IL230	SIS(4.10)
	* *								IL313	SIS(5.13)
	* *								IL67	SIS(5.0)
	* *								IL68	SIS(4.47)
	* *								IL83	SIS(5.53)
	* *								IL91	SIS4.33
	* *								IL99	SIS(4.37)
	*qSIS9*.*8*	9	8.6	S9_8608506	2.17	7.79	-0.05	B	IL116	SIS(4.40)
	* *								IL119	SIS(4.33)
	* *								IL230	SIS(4.10)
	* *								IL99	SIS(4.37)
CHL_SMA	*qCHL11*.*2*	11	2.32	S11_2322899	6.31	15.19	11.73	P	IL350	CHL(45.32)
CHL_LRT	*qCHL3*.*6*	3	6.96	S3_6962390	2.28	4.43	4.87	P	IL219	CHL(22.79)
	* *								IL92	CHL(19.13)
	* *								IL94	CHL(18.03)
	* *								IL98	CHL(44.77)
	*qCHL3*.*25*	3	25.64	S3_25640338	2.48	4.86	4.22	P	IL162	CHL(23.67)
	* *								IL198	CHL(21.31)
	* *								IL98	CHL(44.77)
	*qCHL3*.*26*	3	26.97	S3_26978157	2.06	3.65	2.95	P	IL198	CHL(21.31)
	* *								IL253	CHL(25.62)
	* *								IL336	CHL(21.63)
	* *								IL340	CHL(23.26)
	* *								IL98	CHL(44.77)
	*qCHL11*.*2*	11	2.32	S11_2322899	6.31	12.4	11.73	P	IL350	CHL(45.32)
SHL_SMA	*qSHL1*.*3810*	1	38.1	S1_38108856	2.17	2.64	3.09	P	IL178	SHL(39.27)
	* *								IL323	SHL(44.93)
	* *								IL348	SHL(36.88)
	* *								IL51	SHL(40.27)
	* *								IL89	SHL(38.67)
	*qSHL1*.*3818*	1	38.18	S1_38181791	3.33	5.31	4.34	P	IL178	SHL(39.27)
	* *								IL323	SHL(44.93)
	* *								IL348	SHL(36.88)
	* *								IL51	SHL(40.27)
	* *								IL84	SHL(50.33)
	*qSHL1*.*3863*	1	38.63	S1_38636497	4.4	5.93	4.37	P	IL178	SHL(39.27)
	* *								IL303	SHL(42.05)
	* *								IL323	SHL(44.93)
	* *								IL348	SHL(36.88)
	* *								IL51	SHL(40.27)
	* *								IL84	SHL(50.33)
	*qSHL1*.*3876*	1	38.76	S1_38768787	2.99	4.2	3.63	P	IL137	SHL(35.73)
	* *								IL178	SHL(39.27)
	* *								IL303	SHL(42.05)
	* *								IL348	SHL(36.88)
	* *								IL51	SHL(40.27)
	*qSHL1*.*40*	1	40	S1_40013502	2.81	3.89	2.83	P	IL137	SHL(35.73)
	* *								IL178	SHL(39.27)
	* *								IL206	SHL(33.20)
	* *								IL303	SHL(42.05)
	* *								IL323	SHL(44.93)
	* *								IL348	SHL(36.88)
	* *								IL51	SHL(40.27)
	* *								IL52	SHL(32.40)
	* *								IL65	SHL(37.02)
	* *								IL84	SHL(50.33)
SHL_LRT	*qSHL1*.*3863*	1	38.63	S1_38636497	5.37	21.69	4.59	P	IL178	SHL(39.27)
	* *								IL303	SHL(42.05)
	* *								IL323	SHL(44.93)
	* *								IL348	SHL(36.88)
	* *								IL51	SHL(40.27)
	* *								IL84	SHL(50.33)
	*qSHL8*.*4*	8	4.74	S8_4747595	2.47	9.03	2.98	P	IL106	SHL(36.47)
	* *								IL138	SHL(36.62)
	* *								IL199	SHL(34.03)
	* *								IL271	SHL(34.47)
	* *								IL65	SHL(37.0)
	* *								IL76	SHL(55.80)
SRR_SMA	*qSRR1*.*3818*	1	38.18	S1_38181791	2.08	9	0.39	P	IL178	SRR(4.74)
	* *								IL323	SRR(4.72)
	* *								IL348	SRR(4.35)
	* *								IL51	SRR(4.43)
	* *								IL84	SRR(5.14)
	*qSRR1*.*3863*	1	38.63	S1_38636497	3.04	11.54	0.42	P	IL178	SRR(4.74)
	* *								IL303	SRR(4.89)
	* *								IL323	SRR(4.72)
	* *								IL348	SRR(4.35)
	* *								IL51	SRR(4.43)
	* *								IL84	SRR(5.14)
	*qSRR8*.*5*	8	5.34	S8_5341936	2.22	8.65	0.44	P	IL199	SRR(4.35)
	* *								IL271	SRR(3.98)
	* *								IL65	SRR(4.01)
	* *								IL76	SRR(6.79)
SRR_LRT	*qSRR1*.*27*	1	27.95	S1_27956396	2.54	6.18	0.24	P	IL107	SRR(5.63)
	* *								IL153	SRR(4.00)
	* *								IL160	SRR(3.75)
	* *								IL188	SRR(4.70)
	* *								IL232	SRR(4.04)
	* *								IL238	SRR(4.18)
	* *								IL340	SRR(3.87)
	* *								IL57	SRR(4.33)
	* *								IL70	SRR(4.46)
	* *								IL86	SRR(3.96)
	* *								IL89	SRR(4.83)
	* *								IL93	SRR(3.88)
	*qSRR1*.*2851*	1	28.51	S1_28513474	2.64	6.26	0.28	P	IL107	SRR(5.63)
	* *								IL153	SRR(4.00)
	* *								IL160	SRR(3.75)
	* *								IL188	SRR(4.70)
	* *								IL232	SRR(4.04)
	* *								IL238	SRR(4.18)
	* *								IL340	SRR(3.87)
	* *								IL57	SRR(4.33)
	* *								IL70	SRR(4.46)
	* *								IL51	SRR(4.43)
	* *								IL93	SRR(3.88)
	*qSRR1*.*2853*	1	28.53	S1_28535873	2.07	5.01	0.21	P	IL107	SRR(5.63)
	* *								IL153	SRR(4.00)
	* *								IL160	SRR(3.75)
	* *								IL188	SRR(4.70)
	* *								IL232	SRR(4.04)
	* *								IL238	SRR(4.18)
	* *								IL340	SRR(3.87)
	* *								IL57	SRR(4.33)
	* *								IL70	SRR(4.46)
	* *								IL86	SRR(3.96)
	* *								IL89	SRR(4.83)
	* *								IL93	SRR(3.88)
	* *								IL51	SRR(4.43)
	*qSRR1*.*3863*	1	38.63	S1_38636497	3.91	10.54	0.44	P	IL178	SRR(4.74)
	* *								IL303	SRR(4.89)
	* *								IL323	SRR(4.72)
	* *								IL348	SRR(4.35)
	* *								IL51	SRR(4.43)
	* *								IL84	SRR(5.14)
	*qSRR8*.*5*	8	5.34	S8_5341936	3.16	8.27	0.48	P	IL199	SRR(4.35)
	* *								IL271	SRR(3.98)
	* *								IL65	SRR(4.01)
	* *								IL76	SRR(6.79)
DWT_SMA	*qDWT1*.*41*	1	41.81	S1_41818521	2.07	4.67	0.01	P	IL178	DWT(0.078)
	* *								IL323	DWT(0.087)
	* *								IL52	DWT(0.087)
	* *								IL67	DWT(0.102)
	* *								IL84	DWT(0.133)
	*qDWT1*.*42*	1	42.31	S1_42310908	2.37	5.29	0.01	P	IL178	DWT(0.078)
	* *								IL67	DWT(0.102)
	* *								IL84	DWT(0.133)
	*qDWT7*.*17*	7	17.57	S7_17569558	2.84	6.49	0.01	P	IL153	DWT(0.091)
	* *								IL166	DWT(0.069)
	* *								IL186	DWT(0.082)
	* *								IL188	DWT(0.096)
	* *								IL190	DWT(0.079)
	* *								IL230	DWT(0.111)
	* *								IL303	DWT(0.081)
	* *								IL52	DWT(0.087)
	* *								IL65	DWT(0.102)
	* *								IL84	DWT(0.133)
	* *								IL92	DWT(0.089)
	*qDWT7*.*18*	7	18.8	S7_18801087	2.05	3.98	0.01	P	IL186	DWT(0.082)
	* *								IL188	DWT(0.096)
	* *								IL190	DWT(0.079)
	* *								IL230	DWT(0.111)
	* *								IL232	DWT(0.075)
	* *								IL65	DWT(0.102)
	* *								IL92	DWT(0.089)
	*qDWT7*.*20*	7	20.08	S7_20085299	3.14	7.39	0.01	P	IL186	DWT(0.082)
	* *								IL188	DWT(0.096)
	* *								IL65	DWT(0.102)
	* *								IL84	DWT(0.133)
DWT_LRT	*qDWT1*.*42*	1	42.31	S1_42310908	2.13	7.28	0.01	P	IL178	DWT(0.078)
	* *								IL67	DWT(0.102)
	* *								IL84	DWT(0.133)
	*qDWT5*.*034*	5	0.34	S5_340482	2.26	8.46	0.01	P	IL219	DWT(0.069)
	* *								IL230	DWT(0.111)
	* *								IL313	DWT(0.091)
	* *								IL67	DWT(0.102)
	* *								IL68	DWT(0.096)
	* *								IL83	DWT(0.095)
	* *								IL91	DWT(0.097)
	* *								IL99	DWT(0.082)
	*qDWT7*.*17*	7	17.57	S7_17569558	2.84	11.54	0.01	P	IL153	DWT(0.091)
									IL166	DWT(0.069)
									IL186	DWT(0.082)
									IL188	DWT(0.096)
									IL190	DWT(0.079)
									IL230	DWT(0.111)
									IL303	DWT(0.081)
									IL52	DWT(0.087)
									IL65	DWT(0.102)
									IL84	DWT(0.133)
									IL92	DWT(0.089)

^¥^Parental source of increasing allele: B, Bengal; P, Pokkali.

#### QTLs for shoot Na^+^ concentration

There were no significant QTLs detected for Na^+^ concentration using SSR markers (Table 4). In contrast, QTL mapping using SNP markers detected a single minor QTL located on chromosome 11. The *qNa11*.*5* accounted for 10% of the phenotypic variation in Na^+^ concentration. The Bengal allele at the locus had increasing effect in the shoot Na^+^ ions (Table 5). Therefore, Pokkali allele at this QTL was desirable. Except for IL262, lines containing this QTL with Pokkali allele showed some tolerance despite higher Na^+^ concentration than Pokkali (S3 Table).

#### QTLs for K^+^ concentration

The SMA and LRT methods using SNP markers have both detected a single QTL for K^+^ concentration. The QTL *qK1*.*3863* was mapped on chromosome 1 at the 38.63 Mb region and was responsible for 11% of the variation in K^+^. Allele substitution of Bengal by Pokkali allele had increasing effect of 69 mmolkg^-1^ at the locus (Table 5). In contrast, there were no significant QTLs for K^+^ concentration by SSR markers. Except for IL51, lines containing Pokkali allele at *qK1*.*3863* had higher shoot K^+^ concentration than Pokkali.

#### QTLs for NaK

A single QTL for NaK was detected significant by SMA and LRT. The *qNaK3*.*32* was mapped on chromosome 3 at 32 Mb region. This QTL explained for 11% of the variation in NaK. The Pokkali allele at this QTL had increasing effect. ILs containing this QTL had even higher NaK ratio than Bengal indicating the undesirable effect of Pokkali allele at the locus. On the other hand, there were no QTLs significant in both mapping methods using SSR markers.

#### QTL for SIS

Using SSR markers, the SMA and LRT detected five QTLs for SIS on chromosomes 1, 2, 6, and 7. Three of the QTLs had minor effects (*qSIS2*.*3*, *qSIS6*.*5*, *qSIS7*.*12*) and two QTLs had large effects (*qSIS1*.*39 and qSIS7*.*17*) with a contribution of 10–16% of the SIS phenotypic variation. In contrast to SSR markers, mapping of QTLs by SNP markers detected five significant QTLs for SIS on chromosomes 1, 5, and 9. The *qSIS5*.*034* and *qSIS9*.*8* were significant QTLs in both LRT and SMA methods. However, all QTLs had minor effects, and accounted for only 5–8% of SIS variation. Bengal alleles had increasing SIS effects in all QTLs suggesting desirability of Pokkali alleles at SIS QTLs. Using SSR markers, ILs containing introgressed Pokkali segments at SIS QTLs showed mean SIS of 3.2 to 6.5. In contrast, SIS QTLs by SNP markers included only the ILs with mean SIS of 3.2 to 5.7. Interestingly, ILs containing *qSIS9*.*8* had high tolerance with SIS value not more than 4.4.

#### QTLs for CHL

There was no significant CHL QTL among ILs using SSR markers. However, mapping in ILs by SNP markers detected four QTLs on chromosomes 3 and 11. One of the QTLs (*qCHL11*.*2*) was highly significant with a LOD value of 6.3 and was responsible for 12–15% of the phenotypic variation in CHL content. Introgression of Pokkali alleles had increased CHL effects at QTLs.

#### QTLs for SHL

Six QTLs were detected for SHL by SSR and another six QTLs were detected by SNP markers. The QTLs were located on chromosomes 1, 2, 5, 6, 7, and 8. Using SSR markers, two QTLs were detected on chromosome 1 while SNP markers detected five QTLs in the 38–41 Mb regions. The *qSHL5*.*04* (located at 400 Kb region on chromosome 5), *qSHL6*.*5*, and *qSHL1*.*3863* were highly significant and accounted for 16%, 23%, and 22% of the SHL variation, respectively. The Pokkali alleles at these QTLs had increasing effect for SHL.

#### QTLs for RTL

A single QTL for RTL was significant by SSR markers on chromosome 2. Conversely, there were no QTLs detected by SNP markers in both SMA and LRT methods. The *qRTL2*.*20* accounted for 13% of the RTL variation. Bengal allele at the locus had increasing RTL effect. ILs containing Pokkali allele at *qRTL2*.*20* had a shorter root length under salt stress.

#### QTLs for SRR

Two QTLs located on chromosome 2 and 7 were significant for SSR using SSR markers while SNP markers detected six significant QTLs on chromosomes 1 and 8. The two significant QTLs in SSR mapping (*qSRR2*.*37* and *qSRR7*.*12*) were significant in SMA and LRT methods. Both QTLs had increased effects due to Pokkali alleles and accounted for 13–18% of the SRR variation. In contrast, SNP markers detected only minor-effect QTLs except for *qSRR1*.*3863* that explained 10–12% of the SRR variation. The presence of Pokkali alleles at QTLs had increasing effect on SRR.

#### QTLs for DWT

Four and six significant QTLs were detected by SSR and SNP markers, respectively. The QTLs were mapped on chromosomes 1, 2, 5, 6, and 7. The *qDWT2*.*3* was significant by SMA and LRT and was responsible for 15–28% of the DWT variation. Additionally, the *qDWT7*.*17* accounted for 6–12% of the phenotypic variation while other DWT QTLs had minor effects. Overall, the Pokkali alleles had positive effects in increasing the DWT.

### Comparison of QTLs in ILs and RILs

QTL mapping for seedling salinity tolerance was previously conducted in an F_6_ RIL population developed from a cross involving same parents. S6 Table summarized the additive QTLs detected for the nine traits investigated under salt stress in the RIL population. To validate the QTLs for seedling stage-salinity tolerance, the QTLs detected in RIL and IL populations were compared. Among the 85 QTLs for nine traits mapped in RIL population, 25 QTLs in ILs co-localized or mapped adjacent to 14 QTLs in RIL population (Table 6). For Na^+^ concentration and NaK ratio, there were no significant QTLs detected in the IL population that co-localized to QTLs in the RIL population. For K^+^ concentration, the *qK1*.*3863* was near the *qK1*.*38* in the RIL population. For SIS, a total of five QTLs identified in the RIL population were detected in the IL population including the large-effect *qSIS5*.*1b* which was responsible for 13% of SIS variation in RIL population. For CHL QTLs, both *qCHL3*.*26*, and *qCHL11*.*2* were detected in both populations. For SHL, six QTLs were significant in ILs and co-localized near to *qSHL1*.*38*, that contained the major *sd1* gene for plant height. For RTL, *qRTL2*.*20* was mapped near the region of *qRTL2*.*24*. Six QTLs for SRR identified in IL population were mapped in close proximity of three QTLs detected in the RIL population. Additionally, two QTLs detected in the IL population for DWT were located near *qDWT1*.*40* identified in the RIL population.

**Table 6:** List of significant QTLs detected in Bengal x Pokkali IL (BC_4_F_4_) and F_6_ RIL populations.

Trait	QTLs in IL	Chromosome	Position (Mb)	QTLs in RIL	Interval Position (Mb)
Na^+^ concentration	-				
K^+^ concentration	*qK1*.*3863*	1	38.63	*qK1*.*38*	38.79–39.04
NaK	*-*				
SIS	*qSIS6*.*5*	6	5.40	*qSIS6*.*5*	5.84–5.90
	*qSIS7*.*12*	7	12.80	*qSIS7*.*14*	14.59–14.62
	*qSIS7*.*17*	7	17.50	*qSIS7*.*14*	14.59–14.62
	*qSIS5*.*034*	5	0.34	*qSIS5*.*03*	0.31–0.33
	*qSIS5*.*1*	5	1.47	*qSIS5*.*1b*	1.44–1.47
	*qSIS9*.*8*	9	8.60	*qSIS9*.*8*	8.60–9.07
CHL	*qCHL3*.*25*	3	25.64	*qCHL3*.*26*	26.705–26.709
	*qSHL3*.*26*	3	26.97	*qCHL3*.*26*	26.705–26.709
	*qCHL11*.*2*	11	2.32	*qCHL11*.*2*	2.66–2.72
SHL	*qSHL1*.*3810*	1	38.10	*qSHL1*.*38*	38.28–38.61
	*qSHL1*.*3818*	1	38.18	*qSHL1*.*38*	38.28–38.61
	*qSHL1*.*3863*	1	38.63	*qSHL1*.*38*	38.28–38.61
	*qSHL1*.*3876*	1	38.76	*qSHL1*.*38*	38.28–38.61
	*qSHL1*.*39*	1	39.50	*qSHL1*.*38*	38.28–38.61
	*qSHL1*.*40*	1	40.00	*qSHL1*.*38*	38.28–38.61
RTL	*qRTL2*.*20*	2	20.70	*qRTL2*.*24*	24.961–24.963
SRR	*qSRR1*.*27*	1	27.95	*qSRR1*.*29*	29.561–29.568
	*qSRR1*.*2851*	1	28.51	*qSRR1*.*29*	29.561–29.568
	*qsRR1*.*2853*	1	28.53	*qSRR1*.*29*	29.561–29.568
	*qSRR1*.*3818*	1	38.18	*qSRR1*.*382*	38.28–38.61
	*qSRR1*.*3863*	1	38.63	*qSRR1*.*382*	38.28–38.61
	*qSRR2*.*37*	2	37.60	*qSRR2*.*34*	34.66–35.08
DWT	*qDWT1*.*41*	1	41.81	*qDWT1*.*40*	40.37–40.41
	*qDWT1*.*42*	1	42.31	*qDWT1*.*40*	40.37–40.41

### Analysis of tolerant ILs

The IL population showed normal distribution for the SIS values (S1 Fig). Among 292 ILs, only sixteen ILs with a SIS score of less than or equal to 5.2 were significantly different to the susceptible Bengal parent at α = 0.05. Table 7 summarized the phenotype, and genotype of tolerant ILs. IL84 was the most tolerant line with an average SIS of 3.2 and had low NaK ratio like Pokkali. However, IL84 was morphologically similar to Pokkali in terms of SHL, SRR, and DWT. Among the ILs with mean SIS between 4.0–4.8, IL230 had high shoot K^+^ concentration, low NaK ratio, high CHL, and morphologically intermediate between parents in SHL, RTL, SRR, and DWT. In contrast, IL119 and IL91 were tolerant with SHL similar to Bengal. Other tolerant ILs that showed a SIS of 5.0 to 5.2 had phenotypic attributes intermediate between Bengal and Pokkali under salt stress.

**Table 7:** Phenotypic attributes and genome composition of tolerant ILs.

	Mean phenotypic value under salt stress EC12 dSm^-1^									Line statistics based on 107 SSR markers					Line statistics based on 6797 SNP markers				
BP IL	SIS	Na^+^ (mmolkg^-1^)	K^+^ (mmolkg^-1^)	NaK (ratio)	CHL (SPAD unit)	SHL (cm)	RTL (cm)	SRR	DWT (g)	# of donor segments	# of chr. with segments	% recurrent genome	% donor genome	Chromosomes bearing segments	# of donor segments	# of chr. w/ segments	% recurrent genome	% donor genome	Chromosomes bearing segments
84	3.2	943	599	1.7	21.7	50.3	9.8	5.1	0.133	8	7	86.19	13.81	1, 2, 3, 4, 6, 7, 9	70	11	90.98	9.02	1, 2, 3, 4, 5, 6, 7, 8, 9, 10, 12
230	4.1	1264	601	2.1	25.3	37.7	9.7	3.9	0.111	7	7	85.92	14.08	2, 4, 5, 6, 7, 9, 12	56	10	87.40	12.46	1, 2, 4, 5, 6, 7, 8, 9, 10, 12
119	4.3	1151	426	2.8	22.6	30.7	9.0	3.5	0.075	2	2	95.62	4.38	7, 9	19	5	95.54	4.46	1, 4, 7, 8, 9
91	4.3	1240	412	3.0	21.8	34.5	7.9	4.4	0.097	6	5	92.48	7.52	3, 5, 6, 8, 11,	42	9	94.12	5.88	1, 2, 3, 4, 5, 6, 8, 10, 11,
99	4.4	1062	549	1.9	19.7	33.8	9.3	3.8	0.082	2	2	96.19	3.81	5, 9	30	9	95.95	3.72	1, 2, 4, 5, 6, 7, 8, 9, 10
116	4.4	1185	443	2.7	21.2	33.9	9.3	3.7	0.091	1	1	95.90	4.10	9	27	4	97.51	2.49	1, 3, 9, 11
68	4.5	1167	445	2.7	20.3	36.0	8.3	4.4	0.096	4	4	97.39	2.33	1, 3, 5, 10	21	7	98.55	1.45	1, 2, 3, 4, 5, 8, 10
93	4.5	1254	477	2.6	21.2	37.8	9.9	3.9	0.097	4	4	97.26	2.74	2, 4, 7, 8	55	6	97.26	2.74	1, 2, 4, 5, 7, 8, 9, 11, 12
129	4.7	1309	468	2.8	17.0	35.1	8.3	4.3	0.079	5	4	96.38	3.62	1, 4, 7, 11	43	11	97.45	2.28	1, 2, 3, 4, 5, 7, 8, 9, 10, 11, 12
78	4.8	1104	504	2.2	19.3	34.5	8.6	4.0	0.073	3	3	94.32	5.68	3, 8, 9	19	8	99.41	0.59	1, 2, 3, 4, 6, 8, 9, 11
67	5.0	1148	497	2.4	21.5	35.0	9.0	3.9	0.102	2	2	98.73	1.00	1, 5	21	8	97.63	1.50	1, 2, 3, 4, 5, 7, 8, 11
61	5.1	1022	453	2.3	20.6	31.8	8.7	3.7	0.084	3	3	94.49	4.55	3, 5, 7	20	6	97.05	2.69	1, 2, 3, 5, 7, 8
313	5.1	1424	639	2.2	22.6	35.7	9.4	3.8	0.091	3	3	92.92	7.08	5, 9, 10	32	5	95.70	4.30	1, 5, 8, 9, 10,
65	5.1	1198	475	2.7	19.5	37.0	9.3	4.0	0.102	4	4	95.37	4.63	1, 7, 8, 11	56	11	91.82	8.18	1, 2, 3, 4, 5, 6, 7, 8, 9, 10, 11,
130	5.2	1298	456	3.0	19.8	32.9	8.6	3.8	0.073	6	4	91.84	8.16	1, 3, 7, 8	48	10	97.97	2.03	1, 2, 3, 4, 5, 7, 8, 9, 10, 11
57	5.2	1112	443	2.6	18.5	30.9	7.1	4.3	0.083	1	1	97.36	2.64	11	36	5	95.63	4.37	1, 3, 4, 8, 11
Bengal	7.8	1233	548	2.3	19.0	31.7	8.7	3.7	0.071										
Pokkali	3	941	590	1.6	16.1	47.2	10.0	4.8	0.141										

The number of Pokkali segments detected in each IL ranged between 1–8 segments by SSR or 19–70 segments by SNP markers. On the average, the number of Pokkali segments was 6–12 times higher in case of SNP markers than in SSR. Moreover, the difference in the genome composition of each line by SSR and SNP markers ranged between 1–6%, with higher percentage of recurrent genome detected using SNP markers. Among the sixteen lines, the most tolerant IL84 had the highest number of Pokkali segments and had a genome composition of 86% Bengal and 14% Pokkali by SSR or 91% Bengal and 9% Pokkali by SNP markers. Conversely, IL119 had the lowest number of Pokkali segments and had about 96% Bengal and 4% Pokkali genome composition. Other lines had 1–7 or 19–56 Pokkali segments by SSR or SNP markers, respectively. Many of the tolerant lines had 85–99% of Bengal genome and 1–14% of Pokkali genome.

Lines containing Pokkali alleles at QTLs were indicated in Tables 4 and 5. For simplicity, Table 8 summarized the QTLs contained in each tolerant IL. The IL84 contained three SIS QTLs on chromosomes 1 and 9. Additionally, IL84 was the only line with Pokkali segment at *qK1*.*3863* and it contained Pokkali segments at SHL, SRR, and DWT QTLs on chromosome 1. IL230, on the other hand, had four SIS QTLS on chromosomes 5 and 9 and *qDWT7*.*17* for shoot dry weight. The presence of Pokkali segments at *qSIS9*.*8* and *qSIS9*.*14* had increased salinity tolerance of IL119, IL99, and IL116. However, additional SIS QTLs (*qSIS5*.*034*, *qSIS5*.*1*, *qSIS5*.*2*) in IL99 showed no corresponding decrease in SIS when compared to IL119 and IL116. Except for *qSIS5*.*034* in IL230, IL91, IL99, IL68, IL67, and IL313, there was no other SIS QTL that overlapped among the sixteen ILs. Surprisingly, despite the absence of significant QTLs for nine traits, IL129, IL78, and IL130 showed some tolerance with an average SIS of 4.7 to 5.2. For shoot Na^+^ concentration, Pokkali allele at *qNa11*.*5* had decreasing effect and had a positive effect on salinity tolerance of IL91 compared to IL313 which contained the same SIS QTLs. The Pokkali allele at *qNaK3*.*32* had increasing effect on NaK ratio. Except for IL61, all other ILs had no introgressed segment at *qNaK3*.*32*. Similarly, all of the 16 tolerant ILs had no Pokkali segments at QTLs for CHL. For SHL and SRR, the increasing effect of QTL on chromosome 1 at 38 Mb region was evident in IL84. Likewise, Pokkali segments for DWT QTLs on chromosomes 1 and 7 increased DWT in IL84, IL230, IL 67, and IL65.

**Table 8:** List of tolerant ILs and the QTLs each IL contains.

			QTLs detected by SNP markers					
BP IL	SIS	Na^+^	K^+^	NaK	CHL	SHL	SRR	DWT
**84**	*qSIS9*.*14*, *qSIS1*.*41*, *qSIS1*.*42* *qSIS6*.*5*		*qK1*.*3863*			*qSHL1*.*3818*, *qSHL1*.*3863*, *qSHL1*.*3876*, *qSHL1*.*40*	*qSRR1*.*3818*, *qSRR1*.*3863*	*qDWT1*.*41*, *qDWT1*.*42*, *qDWT7*.*17*
**230**	*qSIS5*.*034*, *qSIS5*.*1*, *qSIS5*.*2*, *qSIS9*.*8*,							*qDWT7*.*17*
**119**	*qSIS9*.*8*, *qSIS9*.*14*							
**91**	*qSIS5*.*034*, *qSIS5*.*1*, *qSIS5*.*2*	*qNa11*.*5*						
**99**	*qSIS5*.*034*, *qSIS5*.*1*, *qSIS5*.*2*, *qSIS9*.*8*, *qSIS9*.*14*							
**116**	*qSIS9*.*8*, *qSIS9*.*14*							
**68**	*qSIS5*.*034*							
**93**							*qSRR1*.*27*, *qSRR1*.*2851*, *qSRR1*.*2853*	
**129**								
**78**								
**67**	*qSIS5*.*034*, *qSIS1*.*41*, *qSIS1*.*42*	*qNa11*.*5*						*qDWT1*.*41*, *qDWT1*.*42*
**61**	*qSIS5*.*2*			*qNaK3*.*32*				
**313**	*qSIS5*.*034*, *qSIS5*.*1*, *qSIS5*.*2*							
**65**						*qSHL1*.*40*, *qSHL8*.*4*	*qSRR8*.*5*	*qDWT7*.*17*
**130**								
**57**	* *	*qNa11*.*5*	* *	* *	* *	* *	*qSRR1*.*27*, *qSRR1*.*2851*, *qSRR1*.*2853*	* *

## Discussion

The ILs showed variation and continuous distribution of the traits, indicating the quantitative nature of salinity tolerance. Several ILs showed transgressive phenotype, suggesting favorable and unfavorable allelic combinations between Bengal and Pokkali (S1 Fig). For shoot Na^+^ and K^+^ concentrations, many ILs accumulated higher Na^+^ ions than Bengal and higher K^+^ ions than Pokkali. However, very few ILs have low NaK ratio. Based on SIS, ILs were skewed toward the Bengal parent and only 16 ILs with mean SIS values less than or equal to 5.2 were significantly different to Bengal (Table 7). Similar to the findings in RILs [19], we did not find a line with higher tolerance than Pokkali in term of SIS. In all SIS QTLs, Pokkali alleles were desirable and had decreasing effect on SIS values. Consistent with the growth response of the RIL population to salinity stress, SHL, RTL, SRR, and DWT were significant and negatively correlated to SIS indicating the negative effect of salt stress on plant’s growth. In contrast, SIS was positively correlated to Na^+^ and K^+^ concentrations in IL population. Overall, the pattern of correlation among traits (Table 2) showed consistency with our previous study in Bengal x Pokkali RIL population [19]. The general relationship among traits in both RIL and IL populations indicated reliable phenotyping. Since the ILs were isogenic to Bengal, the phenotypic deviation of an IL from Bengal could be attributed to the presence of Pokkali segments.

### Genome composition of ILs by SSR and SNP markers

The ILs were genotyped using SSR and GBS derived SNP markers to assess the genomic composition of each IL. Our results showed that 78% of Pokkali genome was transmitted in 72 ILs using SSR markers (Table 3). On average, each line contained three donor segments with 95% Bengal and 5% Pokkali genome (S4 Table). Our result was similar to the results by Tian et al [11] in which the use of SSR markers detected only 68% of the donor *O*. *rufipogon* genome in 159 BC_4_F_4_ lines developed in *O*. *sativa* background. In contrast, GBS-SNP markers indicated 100% transmission of Pokkali genome among 88 ILs. On average, each IL contained 36 Pokkali segments that is equivalent to 4% of rice genome (S5 Table). The use of SSR markers detected 216 introgressed segments while SNP markers detected 3198 segments, a resolution that is fourteen times higher than using SSR markers (Table 3). Majority of the ILs carried 1–2% Pokkali genome with 3-5Mb length based on SSR markers (Fig 2). On the other hand, SNP markers showed that most of the ILs carried 1–3% Pokkali genome of about 100 Kb in length. Furthermore, a total of 18 QTLs were detected for five traits using SSR markers, while 32 QTLs were detected for eight traits by SNP markers (Fig 3). Using SNP markers, at least one QTL was detected for Na^+^, K^+^ concentrations, NaK ratio, and CHL. These results indicated increased efficiency of donor segment detection and higher resolution of mapped QTL using SNP markers. Despite the availability of thousands of SSR markers in rice [34], most QTL mapping studies used less than 200 markers due to low level of polymorphism. Therefore, the low density and sparsity of SSR markers provides less precise information of donor segments and low resolution of QTLs controlling a trait [35]. With the prevalence of SNPs across the rice genome, the increased density of markers proved to be more informative and useful in identifying donor segments and QTLs that were undetected by SSR markers [36]. Nonetheless, both SSR and SNP markers indicated the same average estimate of the percent recurrent genome of ILs (95%), which is very close to the expected 96% of recurrent genome in BC_4_ generation.

### QTLs for traits related to seedling salinity tolerance

Introgression lines are a set of plants containing donor segments in the genetic background of a recurrent parent. The QTLs introgressed in ILs can be considered gain-of- function alleles making it suitable for QTL discovery and verification of previously mapped QTLs [23]. Although numerous QTLs have been detected for traits related to salinity tolerance in rice, firm conclusion on QTLs is still lacking [37]. This is because QTLs are dependent on specific crosses, growth stages from which the populations of plants were characterized [20], and lack of reliable screening methodology. Therefore, validation of QTLs should be done to confirm the utility of QTLs and markers for rice breeding program. Based on our QTL mapping results for salinity tolerance in the RIL and IL populations, Pokkali alleles at SIS QTLs had favorable effects (Tables 4 and 5, S6 Table). Five SIS QTLs detected in RIL population were also significant in IL population (Table 6). The Pokkali allele at *qSIS6*.*5* could lower the SIS score by 1.6 and the most tolerant IL84 carried introgression at this QTL. The large-effect *qSIS5*.*1b* in RIL population clearly contributed to salinity tolerance as indicated by ILs containing this QTL (*qSIS5*.*1*, Table 5). Furthermore, the presence of Pokkali allele at *qSIS9*.*8* in selected ILs showed high level of salt tolerance (ILs with SIS value of 4.0) indicating the usefulness of SIS QTLs due to stability and consistency between populations. In RIL population, *qSIS5*.*1b* was narrowed down to two genes, of which, a gene encoding a lectin protein kinase (LOC_Os05g03450) is a promising candidate. In *A*. *thaliana*, a lectin protein kinase gene was implicated in structural stability of plasma membrane and plant cell wall [38]. In case of *qSIS5*.*034* or *qSIS5*.*03*, a vacuolar ATP synthase (LOC_Os05g01560) was identified as a potential candidate gene [19].

Very few QTLs were detected for Na^+^, K^+^, and NaK ratio in the IL population. The main reason for this is likely due to limited number of ILs included in our QTL mapping. Although we phenotyped 292 ILs, only 72 and 88 lines were actually used in QTL mapping by SSR and SNP markers, respectively. It is possible that some lines carrying introgressions for those QTLs were excluded during optimization of CSSL selection. Nevertheless, we identified QTLs for Na^+^ (*qNa11*.*5*) and NaK ratio (*qNaK3*.*32*) that were not detected in RIL population. The *qNa11*.5 is likely the same as *qSNC11* detected on chromosome 11 by Wang et al. [18]. However, the *qNaK3*.*32* is novel and has not been reported in earlier studies. For shoot K^+^ concentration, *qK1*.*3863* is the same as the *qK1*.*38* detected in RIL population. Pokkali allele at this locus had increasing effect on shoot K^+^ concentration as indicated by increased phenotypic means of the ILs containing this QTL. Lee et al. [39] detected salinity tolerance QTL (*qST1*) around 38 Mb of chromosome 1. The *qST1* was responsible for 26–27% of the variation in salinity tolerance by visual scoring. Consistent to our RIL-QTL mapping, this locus was responsible for about 10 percent of the phenotypic variation for shoot K^+^ concentration. Close to this QTL was a *SNAC2* gene (LOC_Os01g66120) located at 38.39 to 38.40 Mb region of chromosome 1. Transgenic rice overexpressing *SNAC2* gene showed higher germination and growth rate than wild type plants under cold and salinity stres [40].

Previous study indicated the importance of a major QTL *qSKC1* for shoot K^+^ concentration on chromosome 1 [41]. Fine mapping of *qSKC1* led to the cloning of *HKT1;5* gene located at 11.46 Mb region. The gene was implicated in regulating Na^+^/K^+^ homeostasis by unloading Na^+^ ions from xylem for salinity tolerance [42]. In a separate RIL mapping population, *Saltol* QTL for low NaK ratio was identified flanking the region of *qSKC1* [43–44]. Further study on *Saltol* QTL assumed that the same *HKT1;5* gene was responsible for salinity tolerance [17]. Following these results, *Saltol* QTLwas introgressed to local elite varieties in Asia, West Africa, and Russia through marker-assisted backcrossing [45–48]. In our RIL-QTL mapping, QTLs for high shoot K^+^ concentration (*qK1*.*11*) co-localized with low NaK ratio QTL (*qNaK1*.*11*) at 11.52–11.58 Mb region of chromosome 1 (S6 Table). The position of *qK1*.*11* or *qNaK1*.*11* however, is 60Kb downstream of *HKT1;5*. We did not detect any significant QTL near or around *Saltol* or *qSKC1* region in spite of SSR and SNP markers availability at the locus. Among the ILs, IL172 had introgressed Pokkali segment at 10.59–11.62 Mb region flanking the *SKC1/HKT1; 5/ Saltol/qK1*.*11* locus (S7 Table). However, IL172 was very sensitive and had a mean SIS of 8.1. Based on SNP markers, IL172 had 94% Bengal and 6% Pokkali genome composition with 31 introgressed segments distributed on eight chromosomes (S5 Table). While the *Saltol* provided some sort of seedling salinity tolerance, its effect was not validated in our study. Among the 16 most tolerant lines, (Table 8, S7 Table), there was no IL with introgression in *qSCK1/Saltol/ qK1*.*11/qNaK1*.*11* locus. This observation is consistent with the findings of Thomson et al. [17], who identified tolerant lines without the Pokkali allele at the *Saltol* locus in a population of 39 BC_3_F_5_ lines. *Non-Saltol* lines showed minimal differences to *Saltol*-containing lines in salt injury score, NaK ratio, and chlorophyll content. Similarly, Alam et al. [24] did not find significant differences in salinity tolerance based on standard evaluation system (SES) score between *Saltol* and *non-Saltol* QTL-containing backcross lines, thus raising questions on the reliability of *Saltol* to protect rice at the seedling stage from salt stress. Taken together, our results emphasized the importance of other QTLs in the development of salt tolerant rice varieties. Therefore, breeding programs aiming to transfer salinity tolerance to elite local varieties should not be limited to selection of the *Saltol* QTL. Pyramiding of multiple QTLs in addition to *Saltol* may provide unique opportunity of developing salt tolerant varieties.

As indicated by QTLs for SHL, SRR, and DWT, Pokkali allele on chromosome 1 at 38–42 regions have increasing growth effect and could be one of the mechanisms of salinity tolerance. Therefore, seedling vigor under salt stress should also be considered. IL84 had multiple QTLs between 38–42 Mb of chromosome 1. Additionally, IL93, IL65, and IL 57 contained QTLs for SHL, SRR, and DWT and all showed tolerance despite the absence of Pokkali derived-QTLs for SIS, NaK or K^+^ concentration (Table 8). The *qSHL1*.*38* mapped in RIL was responsible for 52% of the variation in SHL and the Pokkali allele at this QTL had an additive effect of 4.5 cm (S6 Table). The stability and increasing effect of *qSHL1*.*38* was confirmed in ILs containing introgression at this region (Table 5). In RIL-QTL mapping study, shoot K^+^ concentration had significant positive relationships to SHL, SRR, and DWT [29]. In this study, IL-QTL mapping results confirmed those relationships by co-localization of *qK1*.*3863* to *qSHL1*.*3863* and *qSRR1*.*3863*. In addition, *qDWT1*.*41* and *qDWT1*.*42* which are adjacent to *qDWT1*.*40* in RIL population also co-localized with *qSIS1*.*41* and *qSIS1*.*42*, respectively. The co-location of different QTLs indicated simultaneous improvement of rice for those traits. For example, introgression of *qK1*.*3863* may increase salinity tolerance. However, this locus will also increase height and SRR, which can make the rice plants susceptible to lodging. Therefore, care should be taken in selecting QTLs for marker-assisted breeding. Overall, fourteen QTLs detected in RIL for K^+^, SIS, CHL, SHL, RTL, SRR and DWT were validated in the IL populations.

### Important QTLs and ILs

Among the tolerant ILs, the most tolerant IL84 behaved like the tolerant Pokkali by accumulating high K^+^ and relatively less Na^+^ in the shoot, resulting in a low NaK ratio. Due to its tall plant stature, dilution of Na^+^ concentration in the leaves could be a possible salt tolerance mechanism in IL84. Additionally, IL84 had medium grain and red pericarp similar to Pokkali. Inspection of the genotypic profile of IL84 (S7 Table) indicated the presence of Pokkali allele for red pericarp gene (LOC_Os07g11020) on chromosome 7 [49]. Therefore, additional backcrossing will be needed to remove these undesirable traits from IL84. Alternatively, the remaining tolerant lines offered salinity tolerance different from Na^+^/K^+^ homeostasis. The 15 ILs had white pericarp and plant height nearly similar to Bengal (Table 7). Despite presence of low K^+^ concentrations in their shoot, these ILs could tolerate high Na^+^ concentrations. Therefore salinity tolerance in these lines is not by Na^+^ exclusion, but more likely by compartmentation of Na^+^ ions in vacuoles and by synthesis of compatible solutes for osmotic adjustment [50]. At this point, the exact mechanism of salinity tolerance is difficult to ascertain. However, based on salinity response, physiological traits, and QTLs contained by tolerant ILs, our results suggest the importance of SIS QTLs in addition to *qSKC1/Saltol/qK1*.*11* for improving salinity tolerance. IL119 is a promising breeding line with similar morphological attributes like Bengal, with high salt tolerance and least number of Pokkali introgression. This line demonstrated the importance of at least two SIS QTLs (*qSIS9*.*8* and *qSIS9*.*14*) contributing to seedling stage salinity tolerance. The IL230 is another breeding line with additional SIS QTLs on chromosome 5. Overall, these selected tolerant ILs offered potential for selection of high yielding version of Bengal with salinity tolerance.

## Conclusion

Consistent with the previous studies, our results indicated complex and polygenic nature of salinity tolerance. In addition to *Saltol* or *qSKC1*, introgression of SIS QTLs should also be considered to improve salinity tolerance through marker-assisted breeding. Due to near-isogenic nature, the tolerant lines identified in this study may serve as improved varieties or donor breeding lines to transfer salinity tolerance to other US varieties. Additionally, the tolerant lines will be useful in fine mapping and positional cloning of genes for salinity tolerance. The SNP markers flanking the QTLs can easily be converted to PCR-based markers for use in marker-assisted breeding.

## Supporting information

S1 Fig Frequency distribution of 292 ILs (BP BC_4_F_4_) for nine traits under salt stress (EC = 12dSm^-1^). B, P, and I indicate the positions of the mean phenotypic values of Bengal, Pokkali, and the IL population. Na^+^ conc., Na^+^ concentration; K^+^ conc., K^+^ concentration; NaK, Na^+^/K^+^ ratio; SIS, salt injury score; CHL, chlorophyll content measured by SPAD-502 unit; SHL, shoot length; RTL, root length; SRR, shoot length to root length ratio; DWT, dry weight.
(TIF)

S1 Table List of SSR markers used in CSSL mapping. (XLSX)

S2 Table List of SNP markers used in QTL mapping among 88 ILs. (XLSX)

S3 Table Phenotypic mean performance of ILs in nine traits under salinity stress. (XLSX)

S4 Table Genome statistics of 72 ILs covering the rice genome by SSR markers. (XLSX)

S5 Table Genome statistics of 88 ILs by SNP markers. (XLSX)

S6 Table Additive QTLs for traits related to seedling-stage salt tolerance in Bengal/Pokkali F_6_ RIL population identified by IM and ICIM methods [19]. (XLSX)

S7 Table Genotype of selected ILs using 6797 GBS-SNP markers. (XLSX)
