# Supplementary figures and images for: Identification and validation of QTLs for seedling salinity tolerance in introgression lines of a salt tolerant rice landrace ‘Pokkali’
Source: PLoS One. 2017 Apr 7;12(4):e0175361. doi: 10.1371/journal.pone.0175361 (PMC5384751; doi:10.1371/journal.pone.0175361)

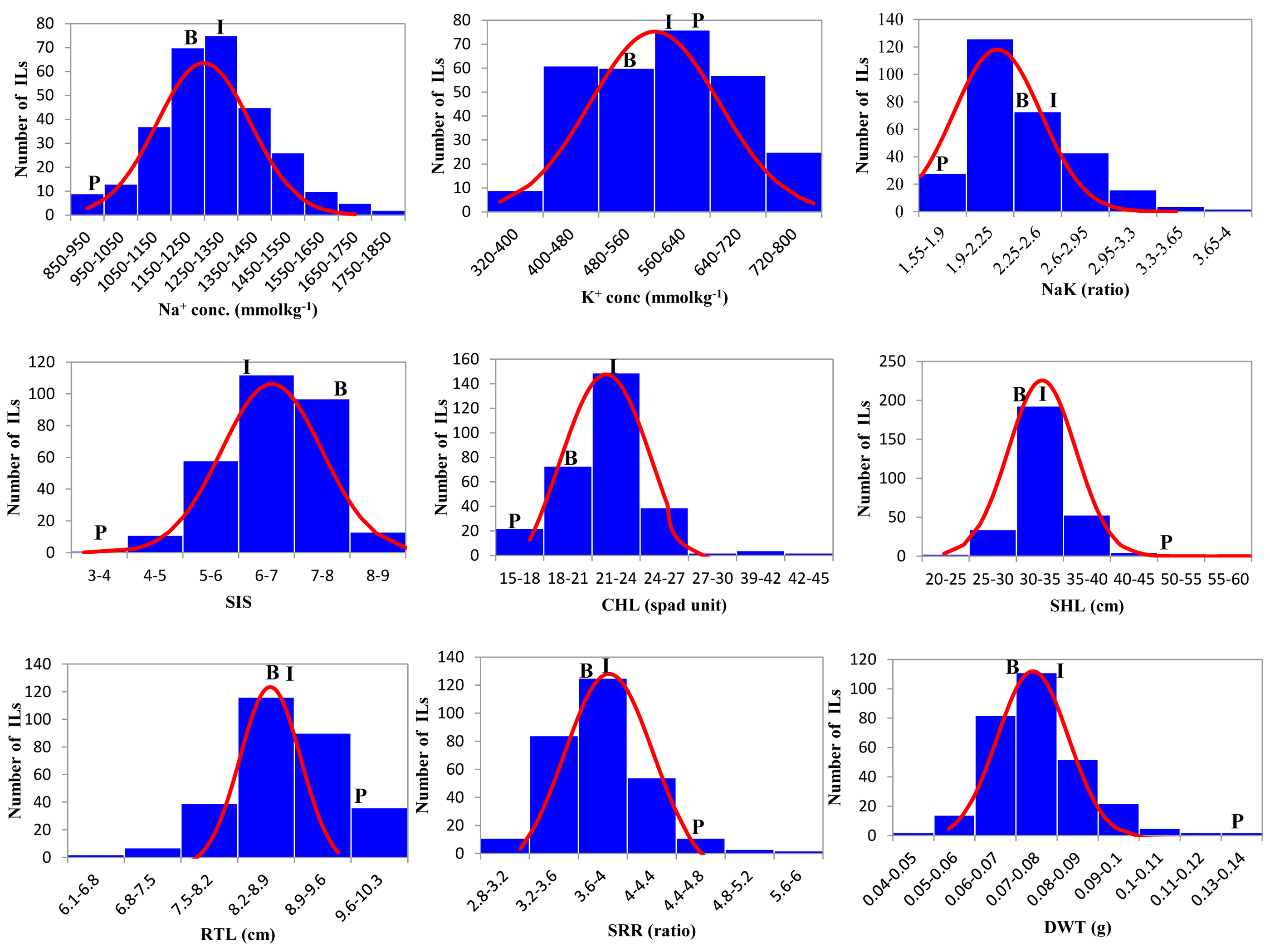

Supplement: S1 Fig — B, P, and I indicate the positions of the mean phenotypic values of Bengal, Pokkali, and the IL population. Na+ conc., Na+ concentration; K+ conc., K+ concentration; NaK, Na+/K+ ratio; SIS, salt injury score; CHL, chlorophyll content measured by SPAD-502 unit; SHL, shoot length; RTL, root length; SRR, shoot length to root length ratio; DWT, dry weight. (TIF) [file pone.0175361.s001.tif]
